# Improvement of SiC Crystal Growth Rate and Uniformity via Top-Seeded Solution Growth under External Static Magnetic Field: A Numerical Investigation

**DOI:** 10.3390/ma13030651

**Published:** 2020-02-01

**Authors:** Minh-Tan Ha, Le Van Lich, Yun-Ji Shin, Si-Young Bae, Myung-Hyun Lee, Seong-Min Jeong

**Affiliations:** 1 Energy and Environmental Division, Korea Institute of Ceramic Engineering and Technology, Jinju-si 52851, Korea; haminhtan.mse@gmail.com (M.-T.H.); shinyj@kicet.re.kr (Y.-J.S.); sybae@kicet.re.kr (S.-Y.B.); mhlee@kicet.re.kr (M.-H.L.); 2 School of Materials Science and Engineering, Hanoi University of Science and Technology, No. 1, Dai Co Viet Street, Hanoi 100000, Vietnam; lich.levan@hust.edu.vn

**Keywords:** silicon carbide, SiC, solution growth, TSSG, external magnetic field, Helmholtz coils, simulation

## Abstract

Silicon carbide (SiC) is an ideal material for high-power and high-performance electronic applications. Top-seeded solution growth (TSSG) is considered as a potential method for bulk growth of high-quality SiC single crystals from the liquid phase source material. The crystal growth performance, such as growth rate and uniformity, is driven by the fluid flow and constitutional flux in the solution. In this study, we numerically investigate the contribution of the external static magnetic field generated by Helmholtz coils to the fluid flow in the silicon melt. Depending on the setup of the Helmholtz coils, four static magnetic field distributions are available, namely, uniform vertical upward/downward and vertical/horizontal cusp. Based on the calculated carbon flux coming to the crystal surface, the vertical downward magnetic field proved its ability to enhance the growth rate as well as the uniformity of the grown crystal.

## 1. Introduction

In the new era of high-power and high-performance electronic devices, the silicon carbide (SiC) substrate plays an important role and can replace the Si substrate in such applications, due to the superior electric and thermal properties of SiC compared to Si [1]. Single-crystal SiC wafers are currently fabricated by physical vapor transport (PVT) [2,3], but their high defect densities are formidable problems in making high-power electric devices. Recently, top-seeded solution growth (TSSG) is gaining attention as an alternative method to grow high-quality SiC single crystals [4,5]. In TSSG, high-purity silicon melts in the graphite crucible, and the carbon from the crucible dissolves to the melt, travels in the medium to the crystal surface, and contributes to crystal growth [6,7]. The TSSG method has a huge advantage in reducing the dislocation density in the crystal by converting and eliminating the threading screw and edge dislocations [8,9].

The TSSG method has the conundrums of low growth-rate and low uniformity of the grown crystal because of low carbon solubility in the molten Si [10], as well as complicated fluid flow in the melt [11,12]. The carbon solubility has been improved by adding some metals in the melt [13,14], but the non-negligible concentration of metal impurities in the grown crystal remains a problem. Meanwhile, a lot of effort has been spent to understand the fluid flow and mass transportation in the melt and to enhance the growth rate. The electromagnetic and Marangoni convections have been confirmed to be the keys that determine the global fluid flow in the melt [7,15,16]. The electromagnetic convection dominates the buoyancy convection defining the velocity field in the melt, while the Marangoni convection causes a strong flux at the edge of the crystal, resulting in instability at the growth-front and reducing the uniformity of the grown crystal. In our previous study [7], we tried to improve the carbon flux to the crystal surface by physically implanting a flow modifier inside the melt and directing the flow as desired. That approach significantly enhanced the growth rate but was less effective in suppressing the Marangoni convection and in improving the uniformity of the grown crystal.

In this study, another approach is numerically investigated in order to improve both the growth rate and the uniformity of the crystal. Different schemes of the external magnetic field generated by Helmholtz coils were applied to the TSSG reactor. The combination of the induction magnetic field and external magnetic fields in the melt, as well as the corresponding velocity fields are derived, and their effects on the crystal growth rate are examined. 

## 2. Description of the Simulation 

**Figure 1:**
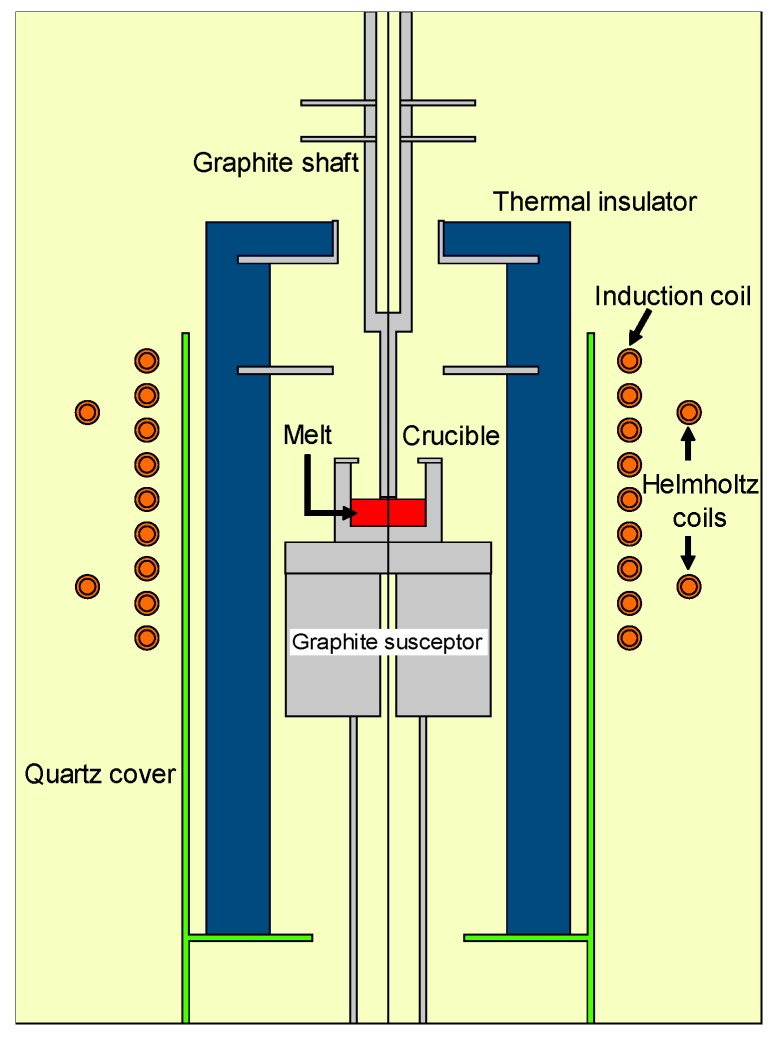
Schematic of the top-seeded solution growth (TSSG) reactor and material domains used in the modeling.

In the present study, the TSSG reactor was composed of a graphite crucible (M501, Morgan, Daegu, Korea), which contained high purity Si chunks (9N, OCI, OCI, Korea, Korea) and was supported by a graphite susceptor, and a graphite shaft (M501, Morgan, Korea) attaching a SiC seed crystal. These parts were enclosed in thermal insulators (Morgan, Korea) and a quartz cover. The reactor was heated up by eddy current in the crucible and susceptor under the time-harmonic magnetic field generated by the induction coil. Figure 1 illustrates the schematic of the practical TSSG system that was used in the simulation. The diameters of the seed crystal, crucible, and induction coil were 1, 5, and 30 cm, respectively. The total length of the induction coil was 18 cm. The height of the melt was 1.7 cm. The center of the induction coil was set at the melt-free surface. In the case of the applied external magnetic field, the Helmholtz coils were inserted outside of the induction coil. The Helmholtz coils included two circular coils, which were placed symmetrically 5.5 cm away from the melt surface, as shown in Figure 1. The diameter of the coils was 38 cm. The setup system was placed in a stainless-steel chamber, which was filled with an Argon atmosphere.

The magnetic fields were obtained by solving Maxwell’s Equations.

(1) ∇ ×H=J

(2) B=∇×A

(3) E=−jωA

(4) J=σE+jωD

where ***H*** was magnetic field intensity (A/m), ***J*** was electric current density (A/m^2^), ***B*** was magnetic flux density (T), ***E*** was electric field intensity (V/m), ***A*** was magnetic potential vector (V·s/m), and ***D*** was electric flux density (V/m). The frequency of the induction coil was set to 8.5 kHz, with an angular frequency ω = 53407 rad/s. In the Helmholtz coils, direct current was applied to avoid eddy currents caused by the Helmholtz coils.

The heat was generated in the graphite parts by total Q of resistive heating $Q_{rh}$ and magnetic loss $Q_{ml}$, which were calculated as:

(5) $$Q_{rh}=0.5real\left(J\cdot E\right)$$

(6) $$Q_{ml}=0.5real\left(i\omega B\cdot H\right)$$

The heat transfer via thermal conduction was calculated as follows:

(7) $$\rho C_{p}\cdot \nabla T=\nabla \cdot \left(k\nabla T\right)+Q$$

where ρ, $C_{p}$, k, and T were density, heat capacity, thermal conductivity, and temperature of the materials, respectively. The heat transfer by radiation at high temperature was included, as described in previous studies [17]. The temperature of the coils was set to 40 °C.

The fluid flow of the silicon melt was composed of several convective flows such as buoyancy, Marangoni, electromagnetic, and forced convective by the crystal rotation. The fluid flow was presumed to be a laminar flow [11]. The Navier-Stokes equation described the fluid flow in the melt:

(8) $$\rho \left(\frac{\partial u}{\partial t}+u\cdot \nabla u\right)=\nabla \cdot \left[-p+\tau \right]+F_{ext}$$

where u was the velocity, p was pressure, $\tau =\;\mu \left(\nabla u+{\left(\nabla u\right)}^{T}\right)$ was the internal viscous stress, and the external volume force $F_{ext}$ was the sum of external force densities such as gravity force and electromagnetic force acting on the melt. The buoyancy convection resulted from non-uniform gravity force density $F_{gra}=\;\rho g$ in the melt, where the density *ρ* was varying in the non-isothermal melt, and g was the gravitational acceleration constant. The electromagnetic convection caused by Lorentz force applied to the melt was $F_{emf}=J\times B$, where J was the current density flow in the melt. In the case without the external Helmholtz coils, $F_{emf}=J\times B_{i}$, where $B_{i}$ was the magnetic field generated by the induction coil. The external magnetic field $B_{e}$ did not contribute to the heating, but played a role in electromagnetic force $F_{emf}=J\times \left(B_{i}+B_{e}\right)$.

The Marangoni effect caused by surface tension depends on the tangential temperature gradient $\nabla_{\tau }T$ at the free surface of the melt [11,16]. The viscous force at the free surface was described by $-p+\tau =\gamma \nabla_{\tau }T$, where γ= −2.5 × 10^−4^ [N/(m·K)] was the temperature-dependent coefficient of the surface tension of silicon-argon. 

At the melt-crystal interface, the fluid velocity was set out of the plane with a value of 30 rpm. The slip condition was applied on the melt surface, as n·u=0 and τ−(τ·n)n=0, where n was a vector normal to the boundary. The no-slip condition u=0 was applied to the solution at the solution-crucible interface. 

The mass transport of carbon in the silicon melt is driven by diffusion and convection, as described by:

(9) ∇·(−D∇c)+u·∇c=0

and the carbon flux was defined by N=−D∇c+u·∇c. The solubility of carbon in silicon $C_{0}=\left(\rho_{Si}/M_{Si}\right)\cdot \left(x_{C}/\left(1-x_{C}\right)\right)$ was derived from the temperature-dependent carbon fraction in molten silicon [10], in which $x_{C}=exp\left(6.249-24460/T\right)$. The equilibrium condition was assumed to be at the interaction interfaces of melt-graphite and melt-crystal; therefore, a boundary condition $C=C_{0}$ was applied to the interfaces of melt-crucible and melt-crystal. A no-flux constraint was applied on the melt surface. The growth rate was calculated by integrating the total carbon flux $F_{c}$ normal to the seed crystal surface, without considering the sticking probability:

(10) $$G=\frac{M_{Si}}{\rho \pi r^{2}}\overset{}{\underset{S}{\iint }}F_{c}dS$$

The properties of materials used in the model are listed in Table 1. The meshing model has been described elsewhere [7,17]. The numerical calculation was conducted using the COMSOL Multiphysics® package, version 5.2, COMSOL Inc., Burlington, MA, USA.

## 3. Results and Discussion

**Figure 2:**
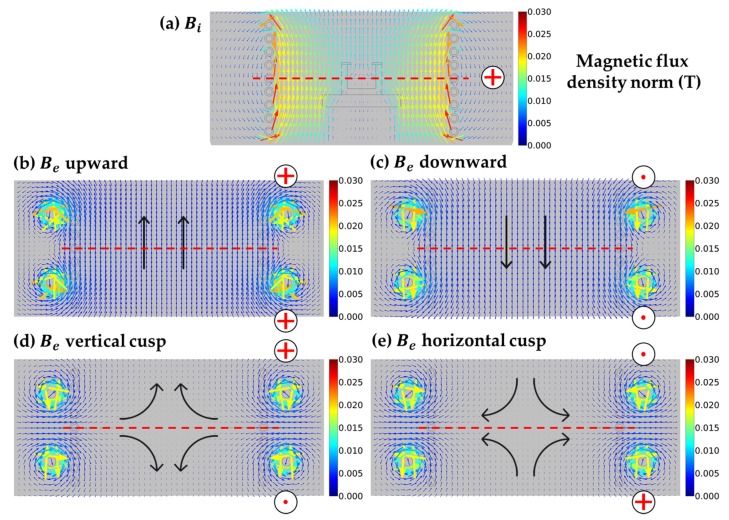
(**a**) The time-averaged magnetic flux density $B_{i}$ generated by the induction coil. The static magnetic field generated by Helmholtz coils with different configurations: (**b**) $B_{e}$ upward, (**c**) $B_{e}$ downward, (**d**) $B_{e}$ with the shape of the vertically directed cusp, and (**e**) $B_{e}$ with the shape of the horizontally directed cusp. The cross and dot symbols indicate the current direction in the coils. The dashed line indicates the center of the coils and the melt-free surface.

The time-averaged magnetic flux density generated by the induction coil $B_{i}$ is shown in Figure 2a. The magnetic field $B_{i}$ was strong inside the coil, and its norm was inversely proportional to the electrical conductivity of the materials, as well as the distance from the coil. In that case, the nominal current in the coil was into the plane, so the direction of the magnetic field was directed upwards. The magnetic fields generated by the Helmholtz coils were dependent on the configuration of input current. If the currents in both coils (upper and lower) are in the same direction as the induction coil current, a uniform upward external magnetic field is formed in the reactor, as shown in Figure 2b, namely, the upward case. If the currents in both coils are in the opposite direction to the induction coil current, the resulting external magnetic field is similar to the upward case but with a downward direction, referred to as the downward case. If the currents in the Helmholtz coils flow in opposite directions to each other, two different configurations of the cusp magnetic field are obtained: vertical cusp or horizontal cusp, as illustrated in Figure 2d,e, respectively.

The center of the cusp magnetic field was a null point, in which the magnetic strength was zero. As shown in Figure 2d, the magnetic field in the melt moved on a conic curve from the crucible wall to the vertical axis of the melt, so it was called the vertically directed cusp or vertical cusp for the sake of brevity. This case could be described as the downward case with a smaller magnitude and an added horizontal component directed towards the center of the melt. Likewise, the horizontal cusp magnetic field in the melt, which is shown in Figure 2e, moved on a conic curve from the bottom of the crucible to the melt-free surface. This case was considered as a weaker version of the upward case with a slightly horizontal component towards the crucible wall added.

**Figure 3:**
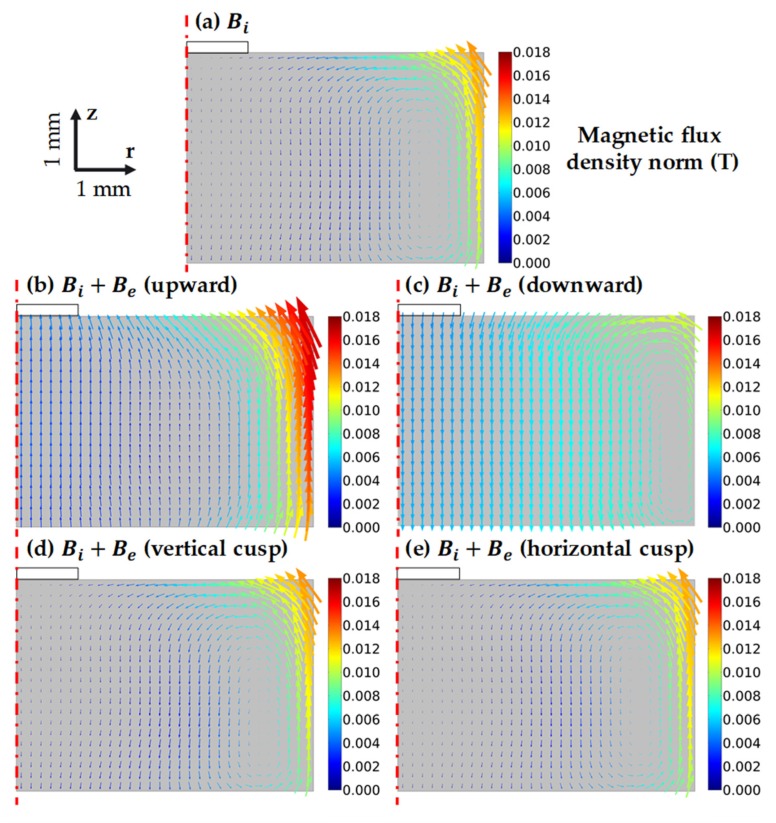
The total magnetic field and magnetic flux density in the melt under the following cases: (**a**) only induction coil $B_{i}$, (**b**) $B_{i}$ and external magnetic field $B_{e}$ upward, (**c**) $B_{i}$ and external magnetic field downward, (**d**) $B_{i}$ and vertical cusp external magnetic field $B_{e}$, (**e**) $B_{i}$ and horizontal cusp external magnetic field $B_{e}$.

Taking a closer look at the magnetic flux density in the melt, the $B_{i}$ was strongest at the melt-crucible interface and rapidly decreased at the center of the melt, as shown in Figure 3a. The maximum at the top corner is about 13 × 10^−3^ T. At the center of the melt, the magnetic density was about 1.7 × 10^−3^ T. Furthermore, $B_{i}$ has a counter-clockwise direction. In the case of $B_{e}$ upward and downward, the external magnetic flux density in the melt was approximately 4.3 × 10^−3^ T. Hence, the $B_{e}$ upward significantly altered the entire magnetic field, as demonstrated in Figure 3b. The total flux was moving upward, and the density at the melt-free surface was enhanced. Meanwhile, the $B_{e}$ downward increased the total flux density at the center of the melt but reduced the flux density at the free surface of the melt. In the case of external cusp magnetic fields, the average magnetic flux densities in the melt were about 0.23 × 10^−3^ T, which were significantly smaller than the $B_{i}$**.** Hence, the external cusp magnetic fields slightly influenced the total magnetic field, as shown in Figure 3d,e.

**Figure 4:**
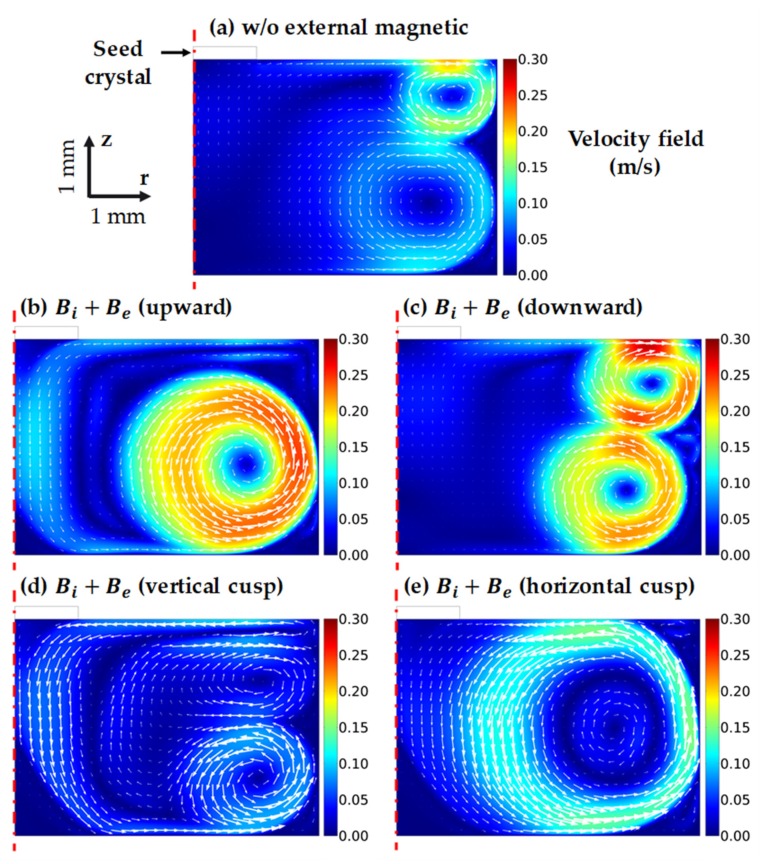
Velocity field [m/s] of the melt in the following cases: (**a**) without external magnetic field $B_{e}$, (**b**) with $B_{e}$ upward, (**c**) with $B_{e}$ downward, (**d**) with $B_{e}$ vertical cusp, (**e**) with $B_{e}$ horizontal cusp.

Figure 4 shows the velocity fields of the melt with and without the external magnetic fields (EMF). The maximum velocity u of the cases was 0.3 m/s, so the Reynolds number Re=ρuL/μ≈ 20,000 was less than the critical Reynolds number of 80,000 for turbulent flow in Si melt [18]. Hence, our presumption of laminar flow at the beginning was correct. At first glance, the fluid flow patterns of the cases without EMF and with $B_{e}$ downward were similar, with two primary vortices. However, the magnitudes of the vortex flow in these two cases were distinguished. Specifically, the intensity of the vortex flow in the case with $B_{e}$ downward was much higher than that of the other case. From that point, we can predict that the average growth rate in the case of $B_{e}$ downward would be higher than the case without EMF. The $B_{e}$ upward case and its weaker horizontal cusp version had the same flow structure with a big vortex near the crucible wall. The fluid flow in the case of the vertical cusp had two vortices, as did the $B_{e}$ downward case, and added a complex motion near the center of the melt. Such fluid flow patterns in Figure 4 proved the critical role of electromagnetic convection in the melt. The increment in the total magnetic field norm led to a significant increase in the velocity magnitude, as in the case of $B_{e}$ upward and downward. Moreover, a slight contribution of the horizontal magnetic component could significantly alter the whole velocity field, as illustrated by the complicated fluid flow in the vertical cusp case and the increment of the diameter of the fluid vortex in the horizontal cusp case.

**Figure 5:**
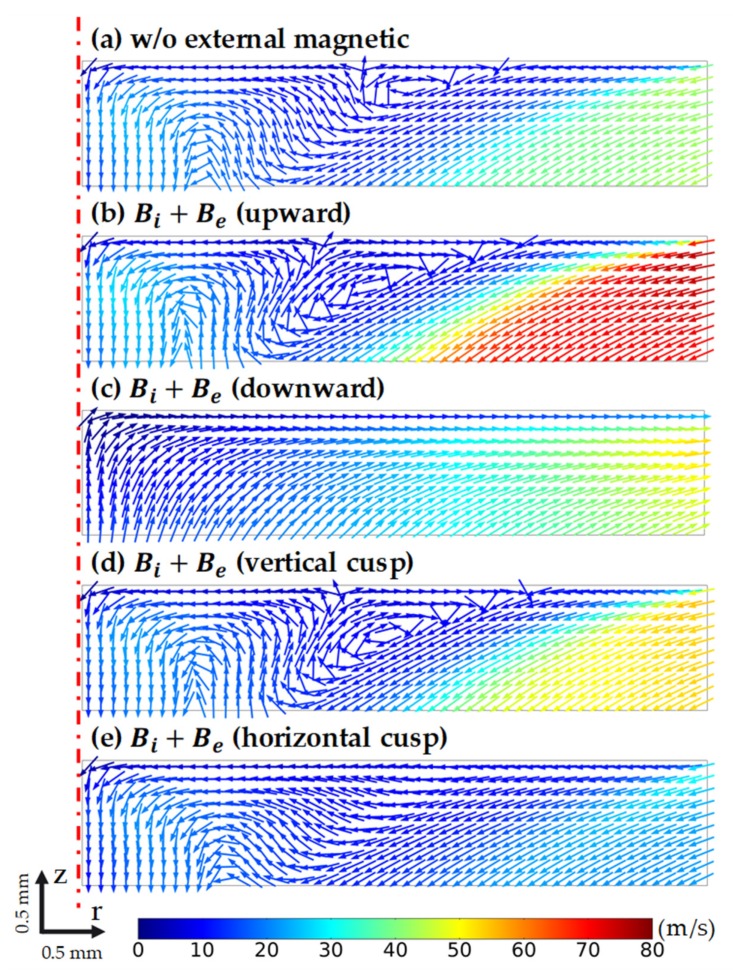
The velocity field in the melt, under the crystal surface, in the cases of: (**a**) without external magnetic field $B_{e}$, (**b**) with $B_{e}$ upward, (**c**) with $B_{e}$ downward, (**d**) with $B_{e}$ vertical cusp, (**e**) with $B_{e}$ horizontal cusp.

The normalized velocity field of the melt under the crystal in different cases is presented in Figure 5. The velocity field in this area could define the uniformity of the grown layer. The flow in the case without EMF showed a soft turbulence at the middle of the crystal surface, and a non-consistent flow direction on the crystal surface. The flow instability was even worse in the case of $B_{e}$ upward and vertical cusp. Only in the case of $B_{e}$ downward and horizontal cusp were the flows consistent in clockwise and counter-clockwise directions, respectively. The capability of controlling the flow direction at the growth front is critical to control the shape/morphology of the grown crystal and to affect the stress field on the crystal and the dislocation conversion efficiency [19]. Moreover, in the case of $B_{e}$ downward, the strong Marangoni convection at the edge of the crystal was totally suppressed without high crystal rotation speed. Therefore, the achievement of a high-uniformity crystal was expected under that condition.

**Figure 6:**
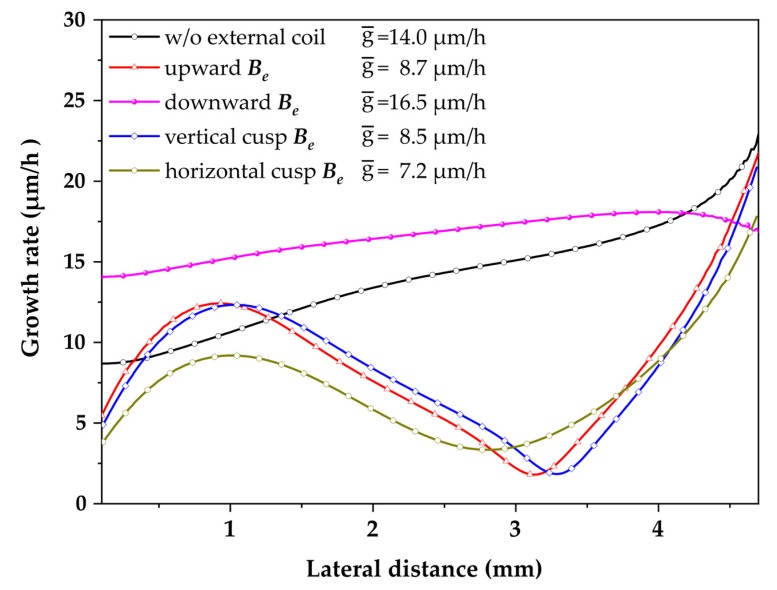
The growth rate in the axial direction from the center to the edge of the crystal in different cases.

The growth rates along the seed crystal under different magnetic fields and at the temperature of approximately 1800 °C are plotted in Figure 6. The average growth rates without the external magnetic field, with $B_{e}$ upward, $B_{e}$ downward, vertical cusp, and horizontal cusp magnetic fields were 14.0, 8.7, 16.5, 8.5, and 7.2 μm/h, respectively. Such growth rates were comparable to the reported data at a similar range of temperature [7,17]. As seen in Figure 6, the growth rates in the case of $B_{e}$ upward and cusp magnetic fields had a low order of uniformity. The growth rates in such cases decreased at the positions of turbulence that happened under the crystal, as illustrated in Figure 6. The growth rate in the case without EMF gradually increased from 8 μm/h at the seed center to 22 μm/h at seed edge (standard deviation σ= 3.3 μm/h). The growth rate in the case of $B_{e}$ downward has much better uniformity with the standard deviation σ= 1.2 μm/h. Such growth rate and uniformity, in this case, agreed with the prediction mentioned in the preceding sections. 

Such results partially agree with those of Wang et al. [20], where the vertical direction magnetic field could enhance the growth rate and the uniformity of grown crystals. Furthermore, the weak cusp magnetic fields were unable to improve such crystal growth performance. However, Wang et al. only reported their work on the vertical upward magnetic field and horizontal cusp magnetic field. Meanwhile, the vertical downward magnetic field showed remarkable enhancement in crystal growth performance.

## 4. Conclusions

Through numerical simulations, a vertical downward external magnetic field, or more precisely a vertical external magnetic field generated by the Helmholtz coils in the antiphase to the induction coil, was proven to be useful in enhancing the carbon transportation from the crucible to the crystal and increasing the growth rate of the SiC crystal. The vertical downward external magnetic field also suppressed the instability caused by Marangoni convection at the edge of the crystal, resulting in a more stable melt flow at the growth front and an improvement in the uniformity of the grown crystal. 

## Figures and Tables

**Table 1:** Properties of materials used in the simulation.

Properties		Ar	Insulator	Graphite	SiC	Si (Melt)
Heat Capacity, *Cp*	[J/(kg∙K)]	520.33	200	710	1400	908.7
Density, *ρ*	[kg/m^3^]	522.21/T	120	1950	3160	3120.45−0.35T
Relative Permittivity, *ε_r_*		1	1	1	1	1
Relative Permeability, *μ_r_*		1	1	1	1	1
Electrical Conductivity, *σ*	[S/m]	1	430	75400	225	1.2 × 10^6^
Thermal Conductivity, *k*	[W/(m∙K)]	0.07	0.336	150∙300/T	61	63
Surface Emissivity, *ε_rad_*		-	0.8	0.9	-	0.3
Dynamic Viscosity, *μ*	[Pa∙s]	-	-	-	-	8 × 10^−4^
Surface Tension coef., *γ*	[N/m∙K]	-	-	-	-	−2.5 × 10^−4^
